# Asylum Medical Work
*Previous articles appeared on August 13, 20, and 27, p. 366.


**Published:** 1921-09-10

**Authors:** 


### THE PROBLEM OF THE INSANE.

### IV.—Asylum Medical Work.*

THERE has been much discussion of the question as to the position and duties of the medical superintendent of asylums. The consensus of opinion seems to be that he is overloaded with administrative work, and has not, therefore, sufficient time left to devote to the purely medical duties. It has been suggested that there should be a lay superintendent as well as a medical one. This division of power might conceivably be carried out successfully. It has not been contended, however, that the medical man has been a failure as an administrator; and it seems wiser, therefore, to continue to have a medical superintendent in general charge. He could easily be relieved of much of the medical work if his senior A.M.O. were made—what he is not now—assistant medical superintendent. At present the latter occupies a rather anomalous position, and the blame for that lies with the visiting committee or with the medical superintendent—or with both. If the deputy were given more power, and at the same time relieved of a great deal of the routine work, he could devote his energies to general medical supervision, referring, of course, important or doubtful matters to his chief. It is unnecessary to go into details, but there seems to be no reason why this should not be a workable scheme.

### ASSISTANT MEDICAL OFFICERS.

The authorities would do well to ponder the suggestions made by Dr. Lomax in regard to the assistant medical officers generally. Conditions, as he notes, have improved, but much remains yet to be done in the matter of pay, facilities for marriage, opportunities for further study, and so on. In many ways the asylum service has not been—nor is it indeed now—one to attract the best men in the profession. Perhaps it will never be, but that is no reason for refraining from the attempt to bring about improvements. With such improvement there will almost certainly be a general amelioration, for, without subscribing to the horrific statements of the alarmists, we may yet agree that much remains to be done. The influence of the medical staff is a potent one even now, and the tone of an institution depends almost entirely upon them. In spite of the critics it is not so greatly a matter of scientific achievement as it is of kindliness and common-sense. Much of the talk to the contrary at the present time is either merely "eye-wash," or an obscuration of plain facts by windy verbiage. It is not intended to depreciate the value of what has been achieved by science and research. It would be futile and mischievous to do so. We owe a great debt to such workers as Mott, Crile, Ford Robertson, John Turner, Orr, and Rows—not to mention foreign researchers. Even casual investigation will reveal to the critics that, in spite of the comparatively unattractive conditions, there are many men working in our asylums to-day who are keenly interested in every aspect of the care and treatment of the insane. There is steady progress; but, if it is desired to hasten the march of events, the public will have to realise that even more money must be spent. Much pathological work is being done, but more is required. The men to do it will be forthcoming when it is made worth their while to take up this branch of science as a career. There is, however, an impediment often overlooked —the objection of relatives to *post-mortem* examinations being performed. This is a matter for the State to deal with.

### EARLIER TREATMENT.

There should also be opportunities for patients receiving earlier treatment apart from certification, and this should apply to all classes of the community. Certification might well be delayed in recent and acute cases, even where the patient has technically passed the borderland, provided, of course, that the patient can be safeguarded from the ministrations of the unskilled or the machinations of the unscrupulous. Hospitals which are provided for the needy but deserving should not be permitted to allocate the greater part of their accommodation to wealthy patients. The private asylums continue to fulfil a need, and there seems no reason why they should not remain for those who are willing and able to pay. It is particularly in regard to them that the Board of Control exercise their vigilance. The Board are usually shot at by those who have no practical knowledge of the work they do and ridiculed as being inefficient. It is safe to say that no one who is cognisant of their immense task, and of the care with which they endeavour to cope with it, will bear the critics out; and, if the business of every Government department were conducted with equal promptitude, we should have a good deal less to grumble about. Instead of being careless they are perhaps at times unduly punctilious, and this may account for some of the odium they have incurred, especially with some people who would be a law unto themselves!

### HELP OR HINDRANCE?

Much good may come of the campaign for the reform of asylums, and let us sincerely hope it will do so. But intemperate abuse and wild, whirling words will not help. The problem must be faced steadily and temperately. In the meantime much harm may be done by unsettling public confidence in all asylums, and a great disservice to those who are striving to obliterate the old and bad traditions by steadily endeavouring to ameliorate the treatment of the insane. Let the critics try to achieve some balance, and not allow themselves to be swayed by prejudice. Above all, let us have practical suggestions and not Utopian schemes.

* Previous articles appeared on August 13, 20, and 27, p. 366.

